# Platinum sensitivity and DNA repair in a recently established panel of patient-derived ovarian carcinoma xenografts

**DOI:** 10.18632/oncotarget.25185

**Published:** 2018-05-15

**Authors:** Federica Guffanti, Maddalena Fratelli, Monica Ganzinelli, Marco Bolis, Francesca Ricci, Francesca Bizzaro, Rosaria Chilà, Federica Paola Sina, Robert Fruscio, Michela Lupia, Ugo Cavallaro, Maria Rosa Cappelletti, Daniele Generali, Raffaella Giavazzi, Giovanna Damia

**Affiliations:** ^1^ Department of Oncology, IRCCS-Istituto di Ricerche Farmacologiche Mario Negri, Milan, Italy; ^2^ Department of Biochemistry, IRCCS-Istituto di Ricerche Farmacologiche Mario Negri, Milan, Italy; ^3^ Medical Oncology Department, Fondazione IRCCS Istituto Nazionale dei Tumori, Milan, Italy; ^4^ Clinic of Obstetrics and Gynecology, San Gerardo Hospital, University of Milan-Bicocca, Department of Medicine and Surgery, Milan, Italy; ^5^ Unit of Gynecological Oncology Research, European Institute of Oncology, Milan, Italy; ^6^ Breast Cancer Unit and Translational Research Unit, ASST Cremona, Cremona, Italy; ^7^ Department of Medical, Surgery and Health Sciences, University of Trieste, Trieste, Italy

**Keywords:** ovarian cancer, patients-derived xenografts, cisplatin, drug resistance, DNA repair

## Abstract

A xenobank of patient-derived (PDX) ovarian tumor samples has been established consisting of tumors with different sensitivity to cisplatin (DDP), from very responsive to resistant. As the DNA repair pathway is an important driver in tumor response to DDP, we analyzed the mRNA expression of 20 genes involved in the nucleotide excision repair, fanconi anemia, homologous recombination, base excision repair, mismatch repair and translesion repair pathways and the methylation patterns of some of these genes. We also investigated the correlation with the response to platinum-based therapy. The mRNA levels of the selected genes were evaluated by Real Time-PCR (RT-PCR) with *ad hoc* validated primers and gene promoter methylation by pyrosequencing. All the DNA repair genes were variably expressed in all 42 PDX samples analyzed, with no particular histotype-specific pattern of expression. In high-grade serous/endometrioid PDXs, the CDK12 mRNA expression levels positively correlated with the expression of TP53BP1, PALB2, XPF and POLB. High-grade serous/endometrioid PDXs with *TP53* mutations had significantly higher levels of POLQ, FANCD2, RAD51 and POLB than high-grade *TP53* wild type PDXs. The mRNA levels of CDK12, PALB2 and XPF inversely associated with the *in vivo* DDP antitumor activity; higher CDK12 mRNA levels were associated with a higher recurrence rate in ovarian patients with low residual tumor. These data support the important role of *CDK12* in the response to a platinum based therapy in ovarian patients.

## INTRODUCTION

Ovarian carcinoma accounts for 2% of all female cancers and is the fifth cause of cancer-related deaths [1]. Epithelial ovarian cancer (EOC) is the most common type (90%) and a recent classification, based on histology, molecular features and natural history, further divides EOCs into two categories [2–4]. Type I EOCs include low-grade serous, low-grade endometrioid, mucinous and clear-cell carcinomas; they are genetically stable, relatively indolent and generally cured by surgery alone and display low chemosensitivity. Type II tumors, the vast majority of EOCs, are high-grade (serous and endometrioid) carcinomas with an aggressive clinical course, genetically unstable and frequently mutated in *TP53*; despite their initial good chemo-sensitivity, their outcome is very poor, with 30% survival after five years. This dismal prognosis is often due to late diagnosis, as symptoms do not generally appear until the disease has already spread outside the ovaries (FIGO stages III/IV), and even if most patients respond to platinum (DDP)-based adjuvant chemotherapy, the majority eventually relapse with a resistant disease [5].

Inactivation of DNA repair is an important oncogenic event in most human cancers [6]. Mutations and/or loss of genes involved in different DNA repair pathways are associated with an increased risk of cancer. However, as most anticancer agents act by damaging DNA, lack/inactivation of these repair pathways renders tumors particularly susceptible to specific chemotherapeutic agents. The striking sensitivity of EOC to platinum-based therapy is thought to be related to underlying defects in homologous recombination (HR) DNA repair, called the *BRCAness* phenotype [7, 8]. Many genetic studies, and more recently The Cancer Genome Atlas (TCGA) project, have shown that high-grade serous ovarian carcinomas are characterized by genetic (germline and somatic mutations) and epigenetic alterations of the HR pathway [9].

Though defective HR is an important mediator of platinum sensitivity in EOC, repair of platinum-induced DNA damage does not involve only the HR pathway [10]. Nucleotide excision repair (NER), fanconi anemia (FA) and mismatch repair (MMR) are all involved in processing platinum-DNA lesions. More than 90% of these lesions are intrastrand cross-links, which are repaired by the NER pathway [11, 12]. Mutations leading to functional inactivation of NER genes lead to extreme platinum sensitivity, as reported in CHO NER mutant cells [13] and in patients with *Xeroderma pigmentosum* or *Cockayne syndrome* [14, 15]. Recently, 8% of high-grade serous EOCs from The Cancer Genome Atlas dataset were shown to have NER alterations, including non-synonymous or splice-site mutations and homozygous deletions of NER genes [16]. Hyper-methylation of *FANCF*, leading to lower protein levels, has been associated with sensitivity to DDP in ovarian cancer cells [17].

While the importance of DNA repair in the extreme sensitivity to DDP has been plainly demonstrated [13], DDP resistance has been less clearly associated with an increase in DNA repair capacity. There are still no validated biomarkers and/or functional assays correlated with tumor DNA repair capacity.

We recently characterized a xenobank of patient-derived ovarian tumor samples (PDXs) and found that these models recapitulate the biological, histological, molecular and pharmacological features of the original human EOC [18]. This EOC-xenobank consists of tumors with different sensitivity to DDP from very responsive, to responsive and resistant tumors, reproducing well the clinical response to therapy in ovarian patients. As DNA repair is an important factor in tumor response to DDP, we analysed the mRNA expression of 20 genes involved in the NER, FA, HR, base excision repair (BER), MMR and translesion repair (TR) pathways and the methylation patterns of some of these genes; subsequently, we investigated the correlations between these data and the response to a platinum-based therapy in our PDX xenobank.

## RESULTS

This study was done on 42 ovarian carcinoma PDXs, whose main characteristics are summarized in Table 1 and Supplementary Table 1.

**Table 1:** Characteristics of the ovarian tumors from which EOC-xenografts derived

	Patient’s original diagnosis				EOC-PDXs			Patient’s original diagnosis				EOC-PDXs	
Xenograft ID	Histotype	Grade	Stage	Source	TP53	DDP response	Xenograft ID	Histotype	Grade	Stage	Source	TP53	DDP response
**MNHOC239**	serous	G2	IV	R	mut		**MNHOC506**	serous	G3	IIIC	n/a^*^	mut	
**MNHOC241**	serous	G2	IC	P	wt	n/a	**MNHOC508**	serous	G3	IIIC	P	mut	
**MNHOC244**	serous	G2	IV	P	mut	n/a	**MNHOC124**	serous/endometrioid	G2	IIIC	P	mut	
**MNHOC250**	serous	G3	IIIC	P	mut	n/a	**MNHOC212**	serous/endometrioid	G2	IIIC	P	mut	
**MNHOC258**	serous	G3	IIIC	P	mut		**MNHOC154**	endometrioid	G2	IIC	R	mut	
**MNHOC266**	serous	G2	n/a	n/a^*^	mut		**MNHOC218**	endometrioid	G3	IIIC	P	mut	
**MNHOC76**	serous	G3	IIIC	R^*^	mut		**MNHOC230**	endometrioid	G3	IIB	R	mut	
**MNHOC18**	serous	G3	IV	P	mut		**MNHOC261**	endometrioid	G2	IIIC	P	mut	
**MNHOC8**	serous	G3	IV	P^*^	mut		**MNHOC78**	endometrioid	G2	IIIC	R	mut	
**MNHOC107**	serous	G3	IIIC	R	mut		**MNHOC109**	endometrioid	G2	IC	R	wt	
**MNHOC111/2**	serous	G3	IIIC	R^*^	mut		**MNHOC503**	endometrioid	G3	IIIA	P	wt	
**MNHOC22**	serous	G3	III	R^*^	mut		**MNHOC145**	endometrioid	G1	IC	P	n.a	n.a
**MNHOC10**	serous	G3	IIIC	P^*^	mut		**MNHOC79**	endometrioid/clear cell	G3	IIIC	R^*^	mut	
**MNHOC8Y**	serous	G3	IV	R^*^	mut		**MNHOC164**	mucinous	G2	IV	P	wt	
**MNHOC84**	serous	G3	IIIC	R	mut		**MNHOC182**	mucinous	G1	IC	P	wt	
**MNHOC106C**	serous	G3	IIIC	R	mut		**MNHOC119**	clear cell	G3	IC	P	wt	
**MNHOC94/2C**	serous	G2	IA	R	wt		**MNHOC142**	clear cell	G3	IIIC	P^*^	mut	
**MNHOC125**	serous	G3	IV	P	mut		**MNHOC135**	mixed mullerian	G3	IIIB	P	mut	
**MNHOC143**	serous	G3	IIIC	P	mut		**MNHOC151**	carcinosarcoma	G3	IIB	P	n.a	n.a
**MNHOC149**	serous	G3	IIIC	P	mut	n.a	**MNHOC9**	not classified	na	IIIC	P	mut	
**MNHOC500**	serous	G3	IIIC	P	mut		**MNHOC88**	undifferentiated	G3	IIIC	R	mut	

Abbreviations: EOC: epithelial ovarian carcinoma; PDXs: patient derived xenografts; P: primary tumor; R: relapse; mut: mutated; wt: wild type; n.a: not available. ^*^Tumor cell suspension transplanted intraperitoneal (ip).

^*^DDP response: *in vivo* activity of DDP as described in Material and Methods. Drug activity was defined as follows: subcutaneous tumors were considered resistant with T/C ≥ 50% (black), responsive with 10 to 50% T/C (dark grey) and very responsive with T/C ≤ 10% (light grey); intraperitoneal tumors were considered resistant with Increase in Life Span (ILS) ≤ 40%, responsive with 40 to 100% ILS, and very responsive with ILS ≥ 100%, according to published criteria.

### mRNA expression of 20 genes involved in different DNA repair pathways

Supplementary Figure 1 shows a heatmap of the expression of the genes analysed in the panel of samples. There was considerable variability in expression levels of the individual genes (Supplementary Table 2) and no histotype-specific cluster was found.

**Figure 1:**
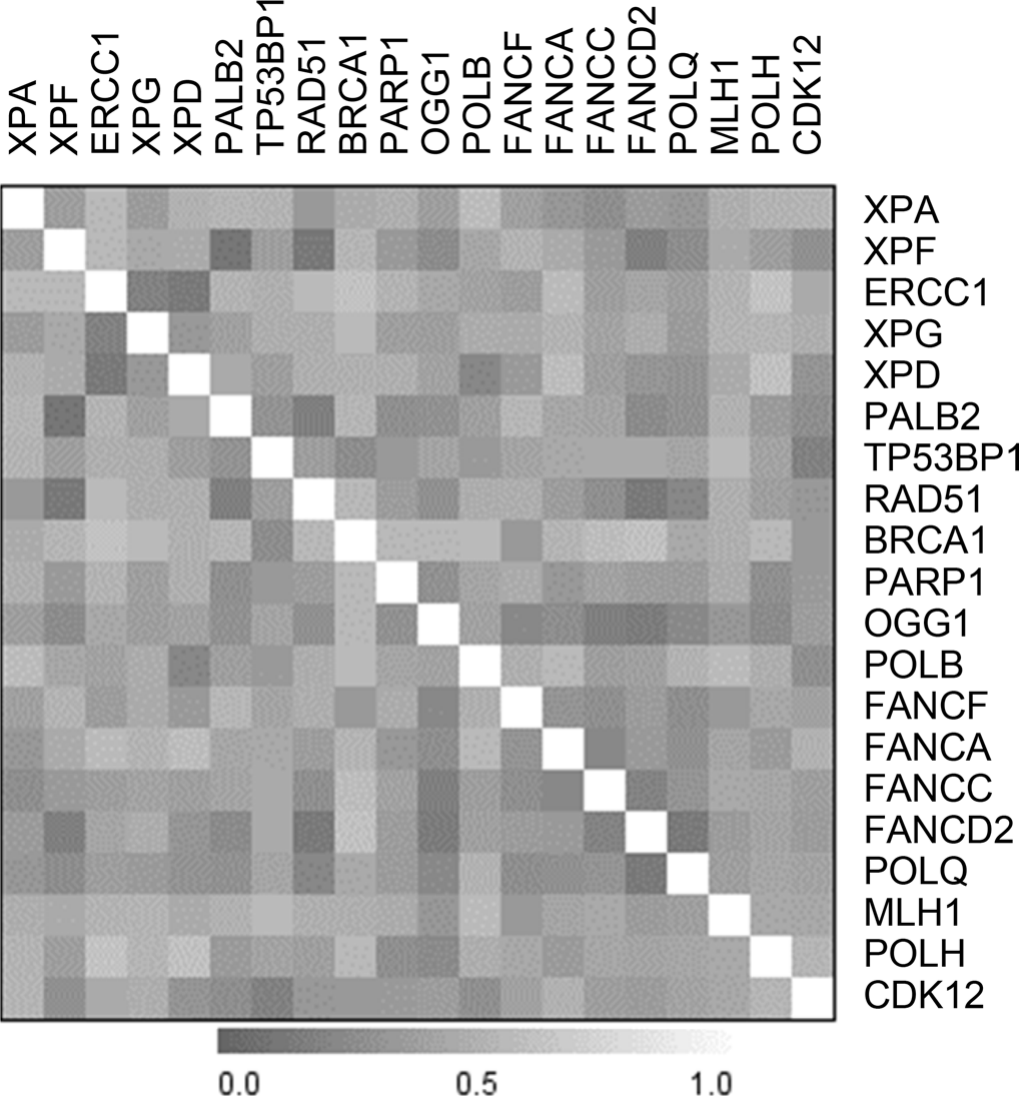
Heatmap of the correlation between single genes’ expression in the subset of high-grade PDXs

Considering that most of the DNA repair pathways explored are multistep processes, we looked for correlations between single gene expression. Supplementary Figure 2A shows the heat-map of correlation and Supplementary Figure 2B reports the correlation indexes with the statistically significant ones red high lightened. As our PDX xenobank was composed of 80% high-grade serous/endometrioid tumors, the most clinically relevant ones, we decided to focus on this more homogeneous subset of tumors and obtained similar results (Figure 1 and Supplementary Table 3). Specifically, the expression of *PALB2*, *FANCC*, *FANCD2*, *OGG1*, *POLQ,* and *RAD51* genes correlated each with the expression of at least six other genes and in some cases genes belonging to the same pathway were inter-correlated (i.e. *FA* genes).

CDK12 mRNA levels positively correlated with the expression of *TP53BP1, PALB2, XPF* and *POLB* genes. We analysed gene expression data from the TCGA database (both from microarrays and RNAseq) and, as detailed in Supplementary Table 4, CDK12 mRNA correlates with three out of four of the transcripts we found in our xenobank (i.e. *TP53BP1, PALB2, XPF)* in both datasets. These data are consistent with the transcriptional role of CDK12 in regulating the expression of some DNA repair genes.

**Figure 2:**
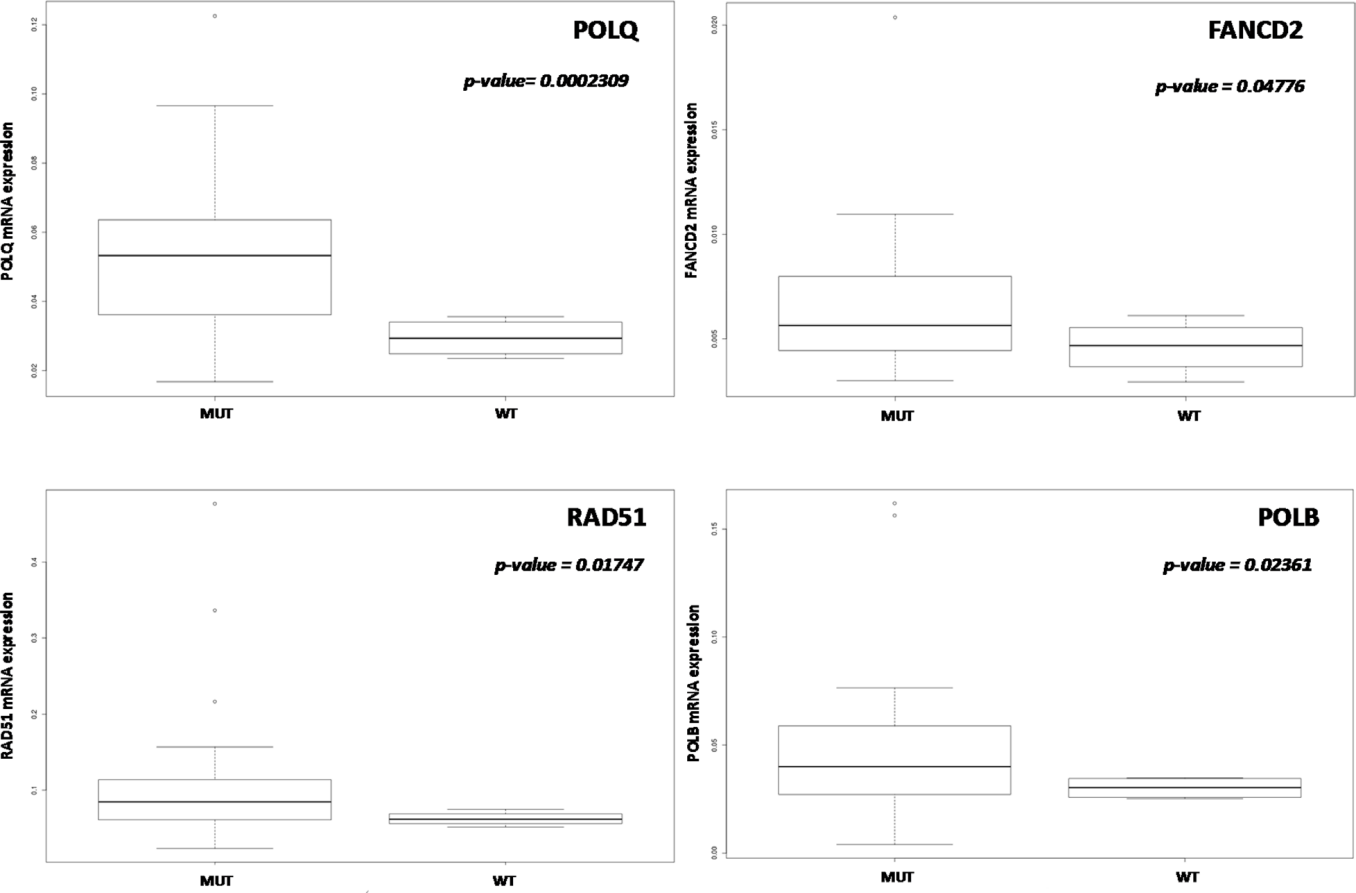
Correlation between *TP53* mutational status and DNA repair gene expression In high-grade ovarian PDXs (*n =* 34) POLQ, FANCD2, RAD51 and POLB mRNA levels were significantly higher in *TP53* mutated PDXs. Data are expressed as median ± standard deviation. *p < 0.05* was considered significant.

No significant differences in gene expression level according to *TP53* mutational status were found when all the tumor types were considered. When we focused on high-grade PDXs, *TP53* mutated xenografts had significantly higher levels of *POLQ*, *FANCD2*, *RAD51*, and *POLB* genes (Figure 2), even these data should be considered with caution considering the sample size (4 wt tumors vs 25 with mutated *TP53*).

### Promoter methylation of *BRCA1, ERCC1, XPA, MLH1, FANCF* and *XPG* genes

**Figure 3:**
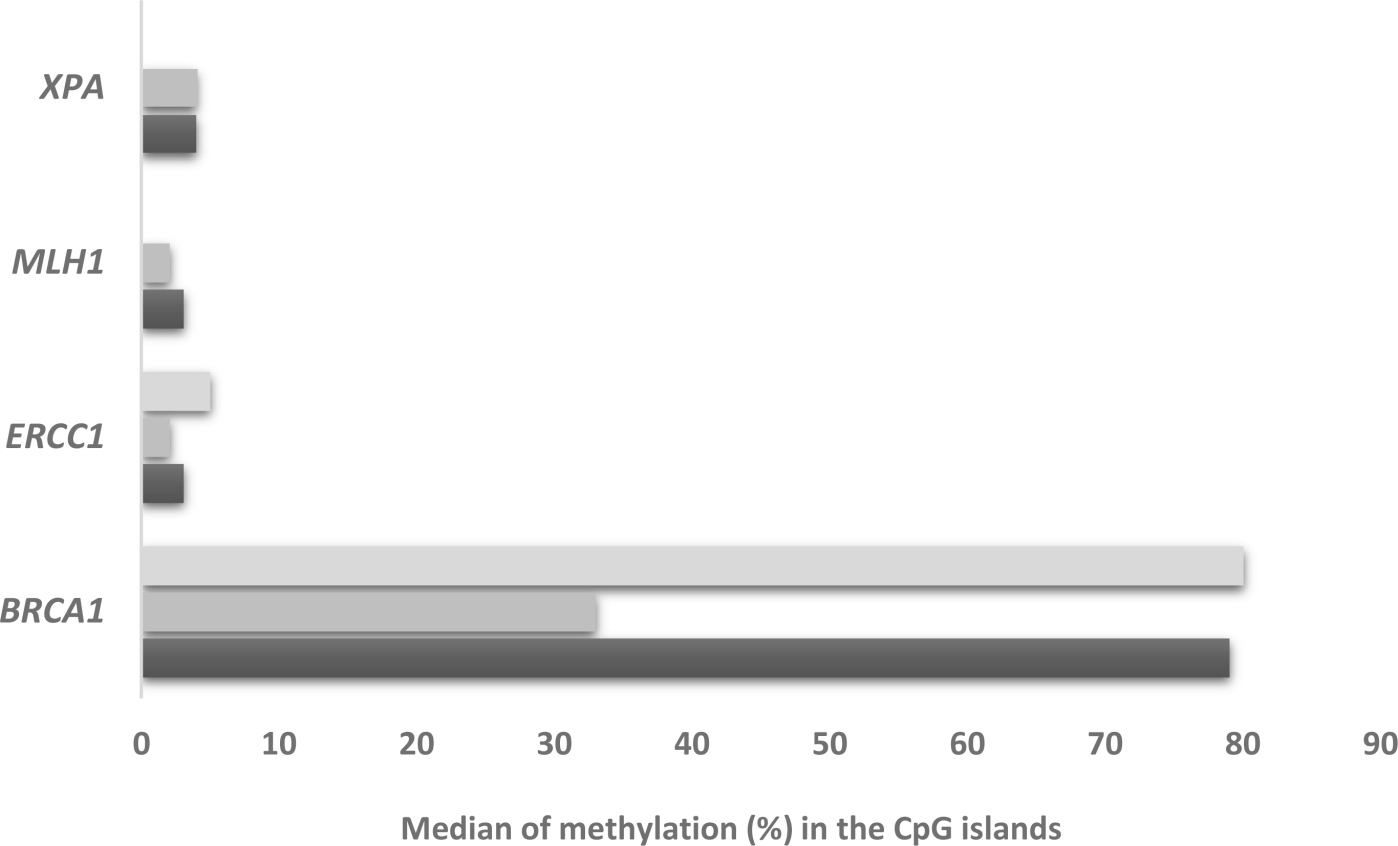
Median % methylation in the CpG islands of *BRCA1, ERCC1, XPA, MLH1* Each bar represents the different CpG islands analyzed.

We then investigated the methylation status of some of the DNA repair genes whose levels were studied. Supplementary Table 5 depicts the percentage of methylation in the selected promoter area of the genes analyzed (see Supplementary Figure 3 and Supplementary Table 6) and Figure 3 reports the median percentage methylation for *BRCA1*, *ERCC1, XPA*, *MLH1* selected regions. *BRCA1* was the most hyper-methylated gene with 51% of the xenografts (20 out of 39) showing all three promoter regions with more than 10% of CpG island methylated. *ERCC1* was scarcely methylated, with only three samples (MNHOC8, MNHOC124 and MNHOC218) showing one promoter region with *>* 10% methylation. Xenograft MNHOC109 was the only tumor which had both *MLH1* promoter regions hyper-methylated, while MNHOC500 presented 11% methylation in only one region; *XPA* promoter was methylated in only one of the selected regions in five out of the 39 xenografts. No methylation of the *XPG* promoter region was detected in any of our PDXs (Supplementary Figure 4), while the *FANCF* promoter regions in MNHOC119 and MNHOC18 PDXs were partially methylated (Supplementary Figure 5). We found no correlation between the *BRCA1* methylation status and its mRNA expression levels (data not shown).

### Gene expression associated to xenografts response to a platinum based therapy

**Figure 4:**
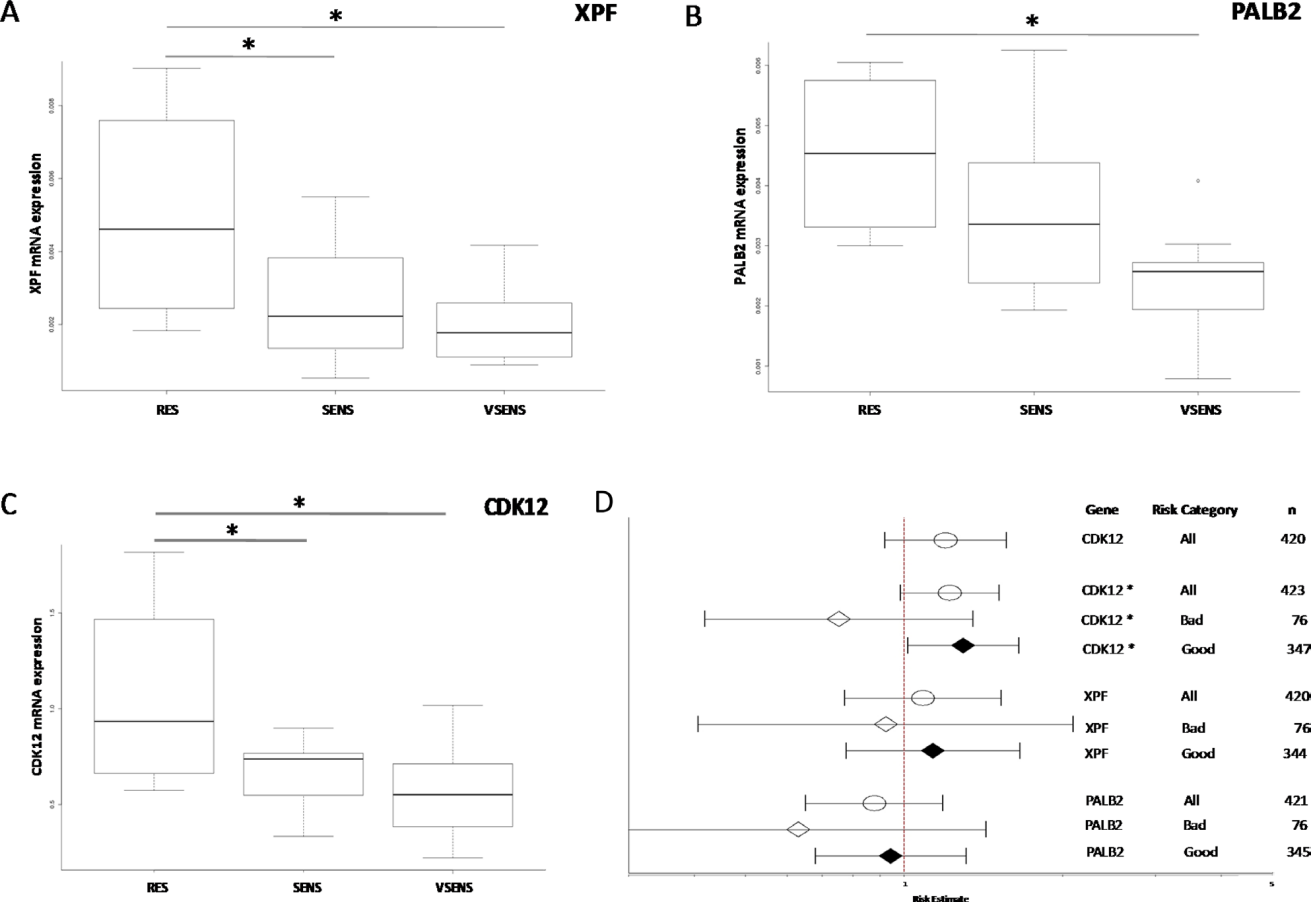
Gene expression levels and DDP response Panels (**A**–**C**): box plots of *XPF*, *PALB2* and *CDK12* gene expression levels respectively and response to DDP treatment in high-grade ovarian cancer PDXs. The circle in the *PALB2* plot indicates an outlier. ^*^p < 0.05. Panel (**D**): forest plot of the relation between gene expression and ovarian cancer patient survival. Estimated risk of recurrence in patients with different mRNA levels of *CDK12*, *XPF* and *PALB2* genes (l- lower CI; -l upper CI; ● estimate). CDK12^*^: the CDK12 mRNA level was taken as zero in patients with *CDK12* mutated; RT (residual tumor) > 2 cm; RT *<* 2 cm.

The relation between the antitumor activity of DDP and mRNA expression levels was investigated with a view to finding possible biomarkers of chemotherapy response. We again focused on high-grade serous and endometrioid xenografts for which an *in vivo* response to DDP was available, and which were classified as very responsive, responsive and resistant (summarized in Table 1 and Supplementary Table 1). The expression levels of three genes (*CDK12* [*p =* 0.017], panel A; *PALB2* [*p =* 0.019], panel B, and *XPF* [*p =* 0.016], panel C) were negatively associated with response to DDP, with higher mRNA levels in resistant xenografts than in responsive ones (Figure 4).

We then looked for independent confirmation in a public database, using the TCGA, where the expression levels of these genes and clinical data, including overall survival, are available. As reported in Figure 4D, patients with higher *CDK12* mRNA levels had a higher risk of recurrence (HR:1.119, 95% CI 0.9188–1.564; *p =* 0.179), though the difference did not reach statistical significance; no association was found for *PALB2* and *XPF* expression levels. The analysis suffered from the fact that patients with *CDK12* mutations were also included. As these mutations have been reported to disable the kinase catalytic activity [19], we considered these patients as having zero protein levels. When the analysis was done on patients stratified for residual tumor (RT) after surgery (more or less than 2 cm) a high level of *CDK12* predicted a worse prognosis for patients with a RT smaller than 2 cm than those with lower *CDK12* levels but also RT *<* 1 cm (HH:1.295, 95% CI 1.016–1.651; *p =* 0.0367) (Figure 4D).

## DISCUSSION

EOC is one of the most chemo-sensitive human tumors, being highly responsive to DDP based front-line therapy; however, relapses with DDP resistant disease occur in most cases. Understanding the molecular basis of the DDP tumor response would be extremely helpful in selecting patients who will benefit most from DDP-based chemotherapy and redirect the less responsive ones to other therapies. Since DNA repair is an important determinant of the cellular response to DDP [20], we studied the expression of 20 genes, and in selected cases their methylation patterns, involved in the repair pathways of platinum-induced DNA damage in a xenobank of EOC-PDXs recently established in our laboratory and in which the response to *in vivo* DDP treatment was available (this manuscript and [18]).

The study showed that: i) all the DNA repair genes were variably expressed in the 42 PDXs analyzed, with no histotype-specific cluster of expression; ii) in high-grade serous/endometrioid PDXs, the CDK12 mRNA levels positively correlated with the expression of *TP53BP1, PALB2, XPF* genes; iii) high-grade serous/endometrioid PDXs with *TP53* mutation had significantly higher levels of *POLQ*, *FANCD2*, *RAD51* and *POLB* genes than *TP53* wt PDXs; iv) except for the *BRCA1* promoter, which was hyper-methylated in 51% of the xenografts, all the other DNA repair gene promoters investigated were scarcely methylated; v) the mRNA levels of *CDK12*, *PALB2* and *XPF* inversely correlated with the *in vivo* DDP antitumor activity; vi) higher CDK12 mRNA levels predicted worse prognosis in patients with residual tumor smaller than 2 cm.

All the DNA repair genes analysed were expressed, but no histotype-specific cluster of expression were found. This might be explained by the samples in our xenobank, where there were only two cases of mucinous and clear cell carcinomas and more than 80% were high-grade serous/endometrioid carcinomas (reflecting the percentage found in clinic). For this reason, we restricted our analysis to this specific sub-group. Interestingly, the expression of genes belonging to the same pathway (i.e. FA and genes involved in the repair of double-strand breaks) were interrelated with positively correlating expression levels, suggesting a common transcriptional control. It was recently reported that DNA repair genes have cell cycle-regulated expression more frequently than average genes, particularly in S phase [21]. While we did not specifically investigate the mitotic index in our PDXs, we collected tumor samples with average tumor masses of 800–1200 mg, when they were in their exponential growing phase.

OGG1 mRNA levels were significantly correlated with genes involved in the removal of intra-strand cross-links and double-strand repair. OGG1 is a DNA glycosylase that removes oxidatively damaged guanines caused by reactive oxygen species–lesions considered poorly cytotoxic, but quite mutagenic [22]. While OGG1 mRNA expression has already been reported to correlate with PARP1 mRNA levels, involved in the same pathway (i.e. BER) [23], no correlation with other DNA repair genes has been reported. These data may underlie a higher than expected cross-talk among the different DNA repair pathways, with *OGG1* as a possible master regulator.

*TP53* mutation is a key event in ovarian tumorigenesis and this mutation is the most frequent one both in early and late stage high-grade serous carcinoma [9, 24]. We found that *TP53* mutated PDXs had higher POLQ, FANCD2, RAD51 and POLB mRNA levels. It has been recently reported that TP53 down-regulates several genes of the FA pathway in many tissues and that loss of TP53 function leads to increased expression of *FA* genes in advanced human cancers, as suggested by the analysis of transcriptomic data in advanced *TP53* mutated human neoplasms (i.e. ovarian adeno-carcinoma, liver and adeno-cortical tumors) [25]. This effect was due to a *TP53*-mediated transcriptional repression through E2F4 binding at *FANCD2* promoter; it remains to be defined whether the same mechanisms apply for *POLQ*, *RAD51* and *POLB* genes. While in murine cell lines the *TP53* dependent down-regulation of FANCD2 was also associated with a decrease in repair activity, it is not yet clear whether higher FANCD2 levels are associated with more DNA repair. This is important as it would imply that higher FANCD2 levels are associated with a higher level of DNA repair and decreased sensitivity to DDP.

DNA repair genes have been found to be variably methylated in human cancers [26, 27]. We found that half of our PDXs showed hyper-methylation in GpC islands of *BRCA1* in the three areas studied (Figure 3 and Supplementary Figure 3); however, this was not correlated with BRCA1 mRNA levels. One explanation might be that these probes, located in the *BRCA1* gene promoter upstream to the transcription site, differ from the ones whose methylation was inversely correlated with *BRCA1* expression in the TCGA [9, 28] and triple-negative breast cancer patients [29]. All the other genes analysed were scarcely methylated, confirming similar findings in ovarian cancer [9, 30]. MNHOC109 PDX with the hyper-methylated *MLH1* promoter was the sample with the lowest MLH1 mRNA (100 times lower than the median, data not shown). Expression of MLH1 has been reported to be inversely correlated with its promoter methylation [31] and resistance to DDP [32]; however, MNHOC109 showed an intermediate sensitivity to DDP (Table 1), suggesting that other factors contribute to DDP’s antitumor activity. Similar consideration should for MNHOC18, in which *FANCF* promoter methylation was associated with low level of mRNA (data not shown) and intermediate responsiveness to DDP.

Our expression data support a potential role of CDK12 in controlling the expression of genes involved in DNA repair (Figure 1). CDK12 is a kinase involved in positively regulating the transcription of genes involved in the DNA damage response, chromosome organization, stress induced gene activation and possibly RNA processing factors [33–35]. The gene has also been found mutated in ovarian cancer and there is evidence that these mutations lead to loss of function, promoting carcinogenesis by impairing homologous recombination repair (HR) and rendering cells particularly susceptible to different anticancer agents, such as PARP inhibitors [19, 36, 37]. In our xenobank, CDK12 levels correlated with the levels of TP53BP1, PALB2, XPF and POLB mRNAs, involved in pathways of repair of double-strand DNA breaks, NER and BER; these genes are also involved in removal of the DDP-DNA damage. Interestingly, CDK12 mRNA levels, with PALB2 and XPF mRNA levels were significantly higher in high-grade PDX models resistant to DDP treatment. When we looked for similar associations between the CDK12, PALB2 and XPF mRNA levels and overall survival (OS) using data from patients treated with platinum therapy in the recent TGCA study in which the corresponding tumor mRNA levels were quantified [9], high PALB2 and XPF mRNA levels were not associated with a worse OS in these patients. We found that high levels of CDK12 were associated with worse OS in patients with a residual tumor after surgery < 2 cm. The data, if confirmed in other cohorts of ovarian patients, will allow the identification of a subgroup of patients (high level of CDK12 mRNA and residual tumor < 2 cm) to be potentially enrolled in clinical trials with alternative therapies. These data partially agree with those recently published in which low CDK12 mRNA levels were associated with improved OS in high-grade ovarian carcinoma [38].

Overall, the present data suggest that the mRNA expression levels of some genes can be important for the response to DDP and they need to be prospectively validated in cohorts of ovarian cancer patients. We are aware that mRNA profiling only captures a subset of cancer genetic changes as other regulation mechanisms are important for gene expression such as microRNAs [39], protein phosphorylation [40] and ubiquitination [41]. One of the hallmark of ovarian cancer is the *BRCAness* phenotype that has been shown to predict sensitivity to both DDP and PARP inhibitors. This phenotype relies on germline/somatic mutations in *BRCA1/2* genes and/or other genes involved in HR (i.e. BROCA-test: exome sequencing to detect mutations in genes encoding proteins involved in HR pathway) [42]; on HDR score that uses a combination of telomeric allelic imbalance, loss of heterozygosity and long segment transition, which all separately associated with *BRCA1/2* mutation in a collective biomarker (i.e. Myriad myChoice-HRD) [43]; on mutational signatures (genomic scars) caused by loss of HR (i.e. BRCA mutations) [44]. Sensitivity to DDP and resistance to PARP inhibitors have been reported to be caused by mutations in genes involved in NER [16], while resistance to both has been seen in *BRCA* mutated patients undergoing reverse mutations on *BRCA* gene restoring the HR activity [45]. We are investigating by whole genome sequencing the mutational signature (s), the presence of telomeric imbalance and loss of heterozygosity in our PDX xenobank to correlate with the response to DDP and these findings will be reported elsewhere.

The validation of functional assays to measure DNA repair activity directly in tumor cells will probably have a more accurate predictive value than the DNA protein expression level. However, this is not easy to accomplish, even if some surrogates [20, 46] and direct tumor measurements of DNA repair capacity have been proposed [47]. We believe our PDXs will be useful for setting up functional DNA repair assays, as suggested by preliminary data on primary cultures and/or organotypic slices to be correlated with the DDP pharmacological activity.

## MATERIALS AND METHODS

### Animals

Female NCr-nu/nu mice obtained from Envigo Laboratories (Italy) were used when six to eight weeks old. Mice were maintained under specific pathogen-free conditions, housed in isolated vented cages, and handled using aseptic procedures. The IRCCS-Istituto di Ricerche Farmacologiche Mario Negri adheres to the principles set out in the following laws, regulations and policies governing the care and use of laboratory animals: Italian Governing Law (D. lg 26/2014; Authorization no.19/2008-A issued March 6, 2008 by Ministry of Health); Mario Negri Institutional Regulations and Policies providing internal authorization for persons conducting animal experiments (Quality Management System Certificate- UNI EN ISO 9001:2008– Reg, N°6121); the NIH Guide for the Care and Use of Laboratory Animals (2011 edition) and EU directive and guidelines (EEC Council Directive 2010/63/UE). The Statement of Compliance (Assurance) with the Public Health Service (PHS) Policy on Human Care and Use of Laboratory Animals was recently reviewed (9/9/2014) and will expire on September 30, 2019 (Animal Welfare Assurance #A5023-01).

### Xenografts

We used a xenobank with 42 recently established ovarian cancer PDXs (30 of them already described in [18]). The study protocol for tissue collection and clinical information was approved by the institutional review board and patients provided written informed consent authorizing the collection and use of the tissue for study purposes. Their histology, *TP53* status and response to *in vivo* DDP treatment are specified in Table 1 and Supplementary Table 1. The majority are high-grade serous/endometrioid PDXs with a mutated *TP53*; the whole spectrum of responses to DDP (very sensitive, sensitive and resistant) is presented ([18] and Supplementary Table 1). DDP antitumor activity was evaluated as previously reported. Specifically, DDP was given i.v. 5 mg/kg 7qx3. Drug activity was defined as follows: subcutaneous (sc) tumors were considered resistant with T/C (mean tumor weight treated/mean tumor weight vehicle treated mice x100) ≥ 50%, responsive with T/C comprises between 10% and 50% and very responsive with T/C ≤ 10%; intraperitoneal (ip) tumors were considered resistant with increase in life span (ILS-median survival time of treated animal/median survival time of vehicle treated mice × 100) ≤ 40%, responsive with ILS from 40% to 100%, and very responsive with ILS ≥ 100%, according to published criteria [48, 49]. High grade serous and endometriod were defined as tumors with histological grade *>* 2.

### RNA isolation and real time-PCR

Tumor samples were obtained from nude mice transplanted sc or ip with ovarian carcinomas; when tumor masses ranged from 800–1200 mg or the abdomen showed signs of ascites, tumor fragments or peritoneal tumor cells recovered by peritoneal lavage were immediately snap-frozen and kept at –80°C until further analysis. Tumor fragments or ascites pellets were homogenized with an Ultra-turrax in RNA lysis buffer in ice and RNA was purified using the SV Total RNA Purification Kit (Promega). Tumor PDX samples were analyzed by real time-PCR to assess the % of murine DNA contamination using primers specifically designed to distinguish human from murine actin. Only samples with more than 70% of human DNA were evaluated. Retro-transcription to cDNA was done using the High Capacity cDNA Archive Kit (Applied Biosystem). The genes selected have a key role in BER (*OGG1, POLB* and *PARP1*), NER (*ERCC1, XPA, XPF, XPD* and *XPG*), and the double strand-break repair pathways (*TP53BP*, *RAD51*, *PALB2*, *BRCA1, FANCA, FANCC, FANCD2* and *FANCF*); in TLR (*POLH*), MMR (*MLH1*), MMEJ (*POLQ*) and in the transcription of some genes involved in the DNA repair activity (*CDK12*). Optimal primer pairs (See Supplementary Table 7) were chosen, spanning splice junctions, using PRIMER-3 software (http://primer3.ut.ee/) and the specificity was verified by detecting single-band amplicons of the PCR products. All the primers were tested for their human specificity using murine cDNA and different proportion of human and murine cDNA (data not shown) and all samples had more than 85% of human RNA. Absolute copy numbers of mRNA were determined by RT-PCR (ABI-7900, Applied Biosystems) with the SYBR Green technique, using an EPMotion 5075 robot (Eppendorf). Standard curves for each gene were included for absolute quantification of mRNA, and normalized as described below.

### Methylation assay

Genomic DNA was extracted from snap-frozen tissues using Maxwel l16 Cell DNA Purification kit (Promega). One microgram of genomic DNA was modified with sodium bisulfite using the Epitect Bisulfite kit (Qiagen) according to the manufacturer’s specifications and as specified in Supplementary Material.

### Data and statistical analysis

PCR data were normalized using the geometric mean of cyclophillin (*CYPA*) and actin (*ACTB*) endogenous controls. The linear correlation between the expression levels of different repair genes was measured by a *Pearson test. Welch t* test was applied to compare two experimental groups (wild type-wt- vs mutated *TP53*). For comparisons of the three groups with different DDP responses, we applied one-way ANOVA, followed by a *Tukey post-hoc test*. High grade PDX sample was 25 for *TP53* mutated and 4 for wt; resistant, sensitive and very sensitive PDXs were respectively 4, 13 and 10. Microarray gene expression data of TCGA ovarian serous cystadenocarcinoma samples and *CDK12* co-expression analysis were retrieved from the cBioPortal platform (http://www.cbioportal.org). RNA sequencing v.2 expression data for a subset of the same database were retrieved from the TCGA data portal (https://tcga-data.nci.nih.gov).

## SUPPLEMENTARY MATERIALS FIGURES AND TABLES




