# Supplementary material for: Platinum sensitivity and DNA repair in a recently established panel of patient-derived ovarian carcinoma xenografts
Source: Oncotarget. 2018 May 15;9(37):24707–17. doi: 10.18632/oncotarget.25185 (PMC5973859; doi:10.18632/oncotarget.25185)
Supplement: Supplementary file 1 [file oncotarget-09-24707-s001.pdf]

Platinum sensitivity and DNA repair in a recently established panel of patient-derived ovarian carcinoma xenografts

SUPPLEMENTARY MATERIALS

For subcutaneously implanted tumors, tumor growth was measured twice a week with a Vernier caliper, and tumor weights (mg = mm^3) were calculated as follows: (length [mm] × width [mm]^2)/2. The efficacy of the treatment was expressed as best tumor growth inhibition [%T/C = (median tumor weight of treated tumors/median tumor weight of control tumors) × 100]. The statistical analysis for assessing DDP antitumor activity relied on the ratio between the experimental (treated mice) and control arm (vehicle-treated mice) area under the curve of the tumor growth divided by the observation time [an adjusted area-under-curve (aAUC)] to quantify tumor growth inhibition. Assuming a coefficient of variation (CV) of aACU equal to 0.60 for both arms, a total of 8-10 mice was required to detect, with a 85% potency, a halving of tumor growth (α-error equal to 10%). For intraperitoneal implanted tumors, criteria for growing tumors were abdominal distension and palpable tumor masses in the peritoneal cavity. Mice were killed when they presented signs of discomfort (survival).

Methylation assay

To verify successful bisulfate modification of the DNA, a region of the Calponin promoter was amplified in the modified DNA sample. For *BRCA1*, *XPA*, *ERCC1* and *MLH1* predesigned primers were used from Qiagen, according to manufacture instruction (Supplementary Table 6 and Supplementary Figure 3), while for *XPG and FANCF and* primers used are reported in Supplementary Table 6 (17, 23). Methylation specific PCR (MSP) was run in a total volume of 25 µL by using Ampli Taq Gold (Applied Biosystems). MPS reactions were subjected to initial incubation at 95°C for 5 minutes, followed by 35 cycles of 95°C for 30 seconds, and annealing at the appropriate temperature for 30 seconds and 72°C for 30 seconds. Final extension was done by incubation at 72°C for 5 minutes. MSP products were separated on 2% agarose gels and visualized with EvaGreen® dye (Jena Bioscience). The PyroMark ID96 instrument and PyroMark ID96 software were used to sequence and analyze the promoter regions of *BRCA1*, *XPA*, *ERCC1* and *MLH1*.

Supplementary Table 1: Antitumor activity of DDP in EOC-PDXs under study

Xenograft ID	Best T/C (day)	DDP response	Xenograft ID	Best ILS (day)	DDP response
**MNHOC239**	17,8 (49)		**MNHOC266***	1136,4* (272)	
**MNHOC258**	36,8 (133)		**MNHOC76***	36*(68)	
**MNHOC18**	21 (36)		**MNHOC8***	84*(62,5)	
**MNHOC107**	17 (85)		**MNHOC111/2***	17* (32)	
**MNHOC84**	36 (66)		**MNHOC22***	224*(88)	
**MNHOC106C**	12 (62)		**MNHOC10***	9* (193)	
**MNHOC94/2C**	36 (28)		**MNHOC8Y***	32* (65)	
**MNHOC125**	10 (105)		**MNHOC506***	117* (89)	
**MNHOC143**	34 (27)		**MNHOC79***	30* (35)	
**MNHOC500**	0,5 (59)		**MNHOC142***	77* (55)	
**MNHOC508**	3 (36)				
**MNHOC124**	3,5 (32)				
**MNHOC212**	0,9 (108)				
**MNHOC154**	38,5 (67)				
**MNHOC218**	10 (53)				
**MNHOC230**	1,2 (105)				
**MNHOC261**	0,5 (59)				
**MNHOC78**	24 (96)				
**MNHOC109**	39 (40)				
**MNHOC503**	44 (116)				
**MNHOC164**	30,4 (36)				
**MNHOC182**	73,8 (49)				
**MNHOC119**	73 (43)		**s.c. (T/C)**	***: IP (ILS)**	
**MNHOC135**	19,7 (81)		T/C ≥ 50	ILS ≤ 40	
**MNHOC9**	14 (74)		10< T/C< 50	40< ILS< 100	
**MNHOC88**	13 (77)		T/C ≤ 10	ILS ≥ 100	

Data are expressed as best T/C (mean tumor weight treated/ mean tumor weight control x 100) for subcutaneous transplanted tumors and increase of life span (ILS-median survival time of treated animal/median survival time of control mice x 100) for intraperitoneally transplanted tumors.

Supplementary Table 2: mRNA expression of the different genes in the panel of EOC-PDXs.
See_Supplementary_Table 2

Supplementary Table 3: Correlations among the expression of the genes studied in the subset of high-grade ovarian PDXs

	MLH1	OGG1	PARP1	POLB	TP53BP1	BRCA1	RAD51	PALB2	POLQ	POLH	FANCA	FANCC	FANCD2	FANCF	XPA	XPD	XPF	XPG	ERCC1	CDK12
**MLH1**	1.00	0.35	0.05	-0.14	-0.12	-0.06	0.01	-0.01	0.22	0.11	0.15	0.11	0.26	0.31	0.03	0.07	0.08	-0.06	-0.04	0.12
**OGG1**	0.35	1.00	**0.41**	0.18	0.16	0.00	**0.41**	**0.41**	**0.52**	**0.49**	**0.45**	**0.65**	**0.69**	**0.53**	0.24	0.18	**0.47**	0.24	0.12	0.25
**PARP1**	0.05	**0.41**	1.00	0.15	0.32	-0.06	0.25	**0.44**	0.27	**0.44**	0.33	0.18	0.25	0.05	0.01	0.02	0.29	0.23	-0.04	0.29
**POLB**	-0.14	0.18	0.15	1.00	0.30	-0.10	0.04	0.23	-0.02	0.02	-0.11	0.24	0.21	0.02	-0.15	**0.56**	0.13	0.05	0.18	**0.43**
**TP53BP1**	-0.12	0.16	0.32	0.30	1.00	**0.50**	0.24	0.38	0.14	0.18	0.10	0.08	0.09	0.16	-0.04	0.24	0.24	0.03	0.02	**0.62**
**BRCA1**	-0.06	0.00	-0.06	-0.10	**0.50**	1.00	-0.10	-0.03	0.03	-0.06	0.01	-0.11	-0.24	0.32	0.12	-0.11	-0.07	-0.12	-0.30	0.27
**RAD51**	0.01	**0.41**	0.25	0.04	0.24	-0.10	1.00	**0.65**	**0.55**	0.22	0.25	**0.44**	**0.72**	0.03	0.31	0.01	**0.72**	0.03	-0.11	0.33
**PALB2**	-0.01	**0.41**	**0.44**	0.23	0.38	-0.03	**0.65**	1.00	**0.41**	0.28	0.13	0.16	**0.48**	-0.08	-0.06	0.09	**0.79**	0.21	-0.05	**0.45**
**POLQ**	0.22	**0.52**	0.27	-0.02	0.14	0.03	**0.55**	**0.41**	1.00	0.13	**0.42**	0.37	**0.71**	**0.45**	0.34	0.38	**0.46**	0.28	0.24	0.17
**POLH**	0.11	**0.49**	**0.44**	0.02	0.18	-0.06	0.22	0.28	0.13	1.00	0.28	0.13	0.16	0.12	-0.04	-0.22	0.24	-0.07	-0.25	-0.07
**FANCA**	0.15	**0.45**	0.33	-0.11	0.10	0.01	0.25	0.13	**0.42**	0.28	1.00	**0.54**	0.29	0.37	0.34	-0.17	0.07	-0.05	-0.14	-0.01
**FANCC**	0.11	**0.65**	0.18	0.24	0.08	-0.11	**0.44**	0.16	0.37	0.13	**0.54**	1.00	**0.59**	**0.44**	**0.45**	0.23	0.29	0.16	0.24	0.24
**FANCD2**	0.26	**0.69**	0.25	0.21	0.09	-0.24	**0.72**	**0.48**	**0.71**	0.16	0.29	**0.59**	1.00	0.26	0.28	0.36	**0.65**	0.04	0.15	0.26
**FANCF**	0.31	**0.53**	0.05	0.02	0.16	0.32	0.03	-0.08	**0.45**	0.12	0.37	**0.44**	0.26	1.00	0.21	0.29	-0.04	0.06	0.25	0.19
**XPA**	0.03	0.24	0.01	-0.15	-0.04	0.12	0.31	-0.06	0.34	-0.04	0.34	**0.45**	0.28	0.21	1.00	-0.01	0.24	0.26	-0.10	-0.05
**XPD**	0.07	0.18	0.02	**0.56**	0.24	-0.11	0.01	0.09	0.38	-0.22	-0.17	0.23	0.36	0.29	-0.01	1.00	0.07	0.33	**0.71**	0.37
**XPF**	0.08	**0.47**	0.29	0.13	0.24	-0.07	**0.72**	**0.79**	**0.46**	0.24	0.07	0.29	**0.65**	-0.04	0.24	0.07	1.00	0.10	-0.11	**0.42**
**XPG**	-0.06	0.24	0.23	0.05	0.03	-0.12	0.03	0.21	0.28	-0.07	-0.05	0.16	0.04	0.06	0.26	0.33	0.10	1.00	**0.61**	0.03
**ERCC1**	-0.04	0.12	-0.04	0.18	0.02	-0.30	-0.11	-0.05	0.24	-0.25	-0.14	0.24	0.15	0.25	-0.10	**0.71**	-0.11	**0.61**	1.00	0.08
**CDK12**	0.12	0.25	0.29	**0.43**	**0.62**	0.27	0.33	**0.45**	0.17	-0.07	-0.01	0.24	0.26	0.19	-0.05	0.37	**0.42**	0.03	0.08	1.00

All the data are expressed as mean of mRNA molecules of three replicated of each PDX.

Supplementary Table 4: Correlations of CDK12 mRNA levels and other DNA repair genes from two different data sets

RNA seq			Affymetrix Microarrays		
Gene	**r to CDK12**	***p*-value**	***Gene***	**r to CDK12**	***p*-value**
BRCA1	0.298019	7.40E-07	***PARP1***	0.26	2.28E-10
TP53BP1	0.286066	2.10E-06	***MLH1***	0.25	1.14E-09
PARP1	0.262365	1.46E-05	***PALB2***	0.22	9.36E-08
POLE	0.260016	1.75E-05	***XPF***	0.2	1.28E-06
XPF	0.240594	7.37E-05	***TP53BP1***	0.19	4.31E-06
POLQ	0.218424	0.000332	***BRCA1***	0.19	4.31E-06
PALB2	0.187205	0.002169	***POLQ***	0.16	0.000113
POLB	-0.17445	0.00432	***RAD51***	0.15	0.000299
ERCC1	-0.16503	0.006987	***XPA***	0.14	0.000746
FANCC	0.1567	0.010482	***FANCD2***	0.13	0.001753
FANCA	0.146949	0.016466	***OGG1***	-0.12	0.003893
FANCD2	0.142286	0.02026			

Supplementary Table 5: Methylation pattern of *BRAC1, ERCC1, MLH1* and *XPA* genes in the PDXs under study

	BRCA1			*ERCC1*			*MLH1*		*XPA*	
**Xenograft #ID**	**Assay-01**	**Assay-02**	**Assay-03**	**Assay -01**	**Assay-02**	**Assay-03**	**Assay-01**	**Assay-04**	**Assay-01**	**Assay-02**
**MNHOC239**	5%	n/a	12%	1%	2%	4%	2%	8%	3%	14%
**MNHOC241**	8%	6%	22%	3%	2%	5%	3%	2%	4%	5%
**MNHOC244**	94%	70%	95%	3%	3%	6%	4%	1%	6%	3%
**MNHOC250**	92%	35%	93%	3%	3%	4%	4%	3%	5%	7%
**MNHOC258**	93%	58%	96%	3%	2%	5%	3%	1%	4%	5%
**MNHOC266**	5%	5%	9%	3%	4%	6%	4%	5%	4%	5%
**MNHOC76**	58%	23%	83%	2%	3%	4%	2%	5%	2%	20%
**MNHOC18**	4%	5%	7%	1%	2%	3%	2%	1%	3%	1%
**MNHOC8**	39%	n/a	73%	0%	2%	15%	2%	3%	1%	n/a
**MNHOC107**	89%	8%	91%	2%	2%	3%	2%	8%	2%	5%
**MNHOC111/2**	93%	73%	82%	2%	3%	5%	3%	1%	3%	3%
**MNHOC8Y**	96%	81%	83%	3%	3%	7%	5%	0%	3%	13%
**MNHOC84**	71%	7%	77%	2%	2%	5%	3%	1%	n/a	6%
**MNHOC106**	94%	70%	84%	3%	3%	4%	3%	5%	5%	6%
**MNHOC94/2C**	1%	3%	5%	2%	2%	4%	2%	1%	4%	1%
**MNHOC125**	96%	69%	94%	3%	3%	5%	3%	4%	4%	5%
**MNHOC143**	3%	2%	5%	2%	1%	8%	2%	0%	3%	0%
**MNHOC149**	6%	4%	29%	2%	2%	3%	2%	2%	3%	3%
**MNHOC500**	86%	55%	79%	3%	2%	5%	3%	11%	4%	4%
**MNHOC506**	79%	n/a	90%	1%	4%	6%	5%	n/a	2%	22%
**MNHOC508**	95%	n/a	84%	2%	n/a	n/a	2%	n/a	2%	4%
**MNHOC124**	95%	82%	90%	3%	3%	19%	4%	3%	5%	7%
**MNHOC212**	95%	71%	92%	3%	3%	6%	5%	2%	4%	12%
**MNHOC154**	90%	33%	91%	3%	2%	5%	3%	2%	4%	4%
**MNHOC218**	96%	59%	90%	3%	5%	18%	3%	3%	4%	5%
**MNHOC230**	94%	41%	90%	3%	2%	4%	3%	3%	5%	4%
**MNHOC261**	64%	30%	72%	4%	2%	5%	3%	2%	4%	5%
**MNHOC78**	3%	3%	6%	2%	2%	4%	3%	2%	4%	3%
**MNHOC109**	6%	5%	31%	3%	2%	8%	96%	87%	4%	0%
**MNHOC503**	15%	n/a	53%	1%	1%	4%	2%	2%	2%	1%
**MNHOC145**	6%	n/a	31%	2%	3%	4%	3%	5%	4%	4%
**MNHOC79**	90%	46%	91%	1%	1%	0%	1%	2%	3%	0%
**MNHOC164**	92%	37%	77%	4%	5%	5%	4%	1%	4%	8%
**MNHOC182**	41%	12%	77%	1%	1%	5%	4%	3%	2%	3%
**MNHOC119**	93%	6%	82%	2%	1%	4%	3%	0%	3%	3%
**MNHOC142**	47%	35%	80%	4%	3%	5%	3%	3%	2%	2%
**MNHOC135**	83%	46%	82%	4%	3%	8%	4%	3%	6%	8%
**MNHOC151**	5%	3%	13%	2%	2%	3%	2%	1%	3%	3%
**MNHOC9**	14%	10%	50%	4%	3%	4%	3%	2%	3%	3%

n/a: not available.W

Supplementary Table 6: List of the methylation probes and primers used in the present study

Gene	Assay name	Qiagen Assay Name	Number of CpG sites included
BRCA1	*BRCA1-01*	Hs_BRCA1_01_PM PyroMark CPG assay PM00064862	4
BRCA1	*BRCA1-02*	Hs_BRCA1_02_PM PyroMark CPG assay PM00064869	3
BRCA1	*BRCA1-03*	Hs_BRCA1_03_PM PyroMark CPG assay PM00064876	4
ERCC1	*ERCC1-01*	Hs_ERCC1_01_PM PyroMark CPG assay PM00071904	6
ERCC1	*ERCC1-02*	Hs_ERCC1_02_PM PyroMark CPG assay PM00071911	4
ERCC1	*ERCC1-03*	Hs_ERCC1_03_PM PyroMark CPG assay PM00185682	5
MLH1; EPM2AIP1	*MLH1-01*	Hs_MLH1/EPM2AIP1_01_PM PyroMark CPG assay PM00104839	5
MLH1; EPM2AIP1	*MLH1-04*	Hs_MLH1/EPM2AIP1_04_PM PyroMark CPG assay PM00104860	6
XPA	*XPA-01*	Hs_XPA_01_PM PyroMark CPG assay PM00142212	6
XPA	*XPA-02*	Hs_XPA_02_PM PyroMark CPG assay PM00142219	5

Gene	Primer Name	Sequence (5′ -> 3′)
FANCF	FANCF Met F	TTTTTGCGTTTGTTGGAGAATCGGGTTTTC
	FANCF Met R	ATACACCGCAAACCGCCGACGAACAAAACG
	FANCF UnMet F	TTTTTGTGTTTGTTGGAGAATTGGGTTTTT
	FANCF UnMet R	ATACACCACAAACCACCAACAAACAAAACA
XPG	XPG Met F	GCGGATTTATTAGCGAAGGCGG
	XPG Met R	CACTAATAAAAACGCATTAAAACGAA
	XPG UnMet F	TTTGTGGATTTATTAGTGAAGGTGGG
	XPG UnMet R	ATAAAAACACATTAAAACAAAAAAAC

Supplementary Table 7: List of the primers used for the RT-PCR of the studied genes

Function	Gene	Primer F	Primer R
Housekeeping	***ACTIN***	TCACCCACACTGTGCCCATCTACGA	CAGCGGAACCGCTCATTGCCAATGG
Housekeeping	***CYCLO A***	GACCCAACACAAATGGTTCC	TTTCACTTTGCCAAACACCA
Mismatch repair (MR)	***MLH1***	AAGCCATGTGGCTCATGTTA	AGGGGCTTTCAGTTTTCCAT
Base Excision repair (BER)	***OGG1***	CTCCACTCCTGCCCTGTG	CCAGTGTGCAGGACTTTGC
Base Excision repair (BER)	***PARP***	AAGAAATGCAGCGAGAGCAT	CCAGTGTGGGACTTTTCCAT
Base Excision repair (BER)	***POLB***	TGCCTGGAGTAGGAACAAAAA	GGAAATTGATGGATGAACTCG
Double strand break repair	***TP53BP***	TGGTTCCATCAGTCAGGTCA	ACAGCAGGAGCAGATTCCAC
Double strand break repair	***POLQ***	GCTGGAACTTTTGCTGACCA	TCATGCCAACGATTTGCACA
Homologous Recombination (HR)	***BRCA1***	GAACGGGCTTGGAAGAAAAT	GTTTCACTCTCACACCCAGA
Homologous Recombination (HR)	***RAD51***	CAGATGCAGCTTGAAGCAAA	TTCTTCACATCGTTGGCATT
Homologous Recombination (HR)	***PALB2***	CTTGGCAGTGGGAAAAACTT	TTCCCAAAGCTACACACACG
Translesion synthesis	***POLH***	CTGGCACAAGTTCGTGAGTC	CGTTCAATCACAGCAAAACG
Fanconi Anemia (FA)	***FANCA***	GAGACCAGTCACCCTGTGCT	CAGAAGGAAAGACGGGAGAA
Fanconi Anemia (FA)	***FANCC***	GGCAAAAGCTTGTTGGAATC	CCAGGAGTTAAGTTTTGATTGTCC
Fanconi Anemia (FA)	***FANCD2***	CCTCGACTCATTGTCAGTCAAC	GATGATGTCATGCTGCAGGT
Fanconi Anemia (FA)	***FANCF***	GCTAGTCCACTGGCTTCTGG	GGTGGCGGCTAGTCACTAAA
Nucleotide excision repair (NER)	***XPA***	ATGCGAAGAATGTGGGAAAG	CTTGTTTTGCCTCTGTTTTGG
Nucleotide excision repair (NER)	***XPD***	GTGGCCATCAGCTCCAAAT	CAGCAGGAGGTTCCCATAGT
Nucleotide excision repair (NER)	***XPF***	TTGTGAGGAAACTGTATCTGTGG	AGCAAGCATGGTAGGTGTCA
Nucleotide excision repair (NER)	***XPG***	TCTGGAAGCTGCTGGAGTG	GACAAAAGGAATGGCAGGAG
Nucleotide excision repair (NER)	***ERCC1***	CCAACAGCATCATTGTGAGC	TCTTGGCCCAGCACATAGTC
Transcription kinase	***CDK12***	TTGTCACAGATAAACAAGATGCAC	TGCACCAAACCAGATTCTAGC

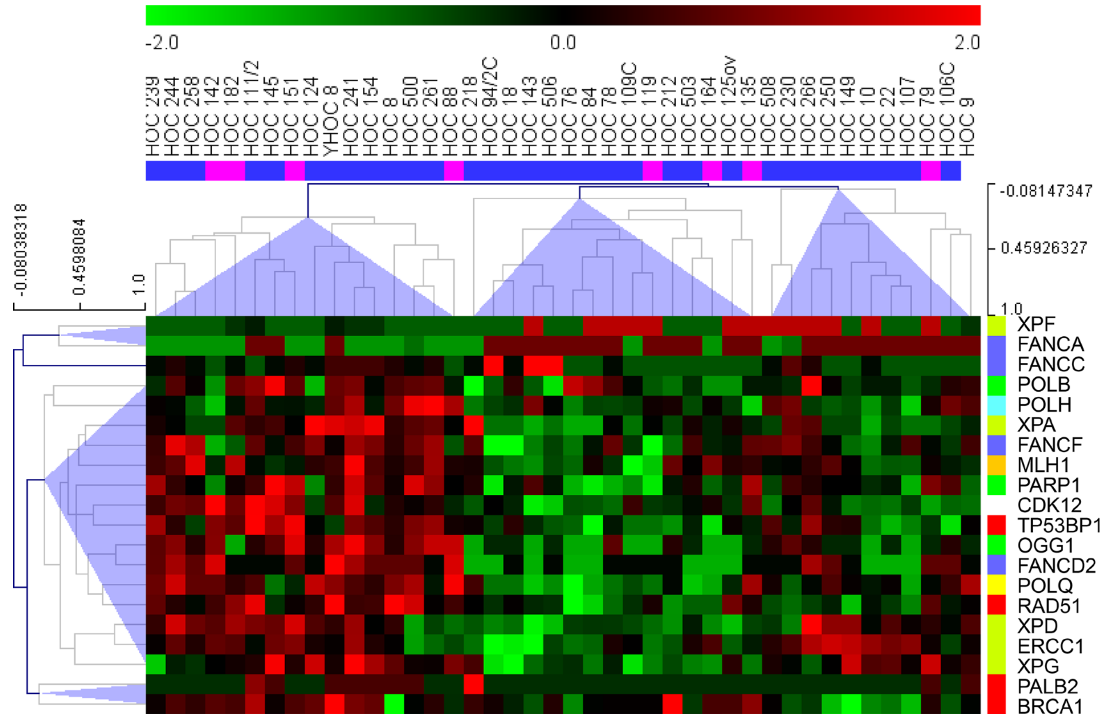

Supplementary Figure 1: Expression pattern of DNA repair genes in the 42 EOC PDXs. Heatmap showing the expression of 20 genes involved in the main DNA repair pathways (rows) analyzed by RT-PCR in the 42 ovarian cancer PDXs (columns).

A

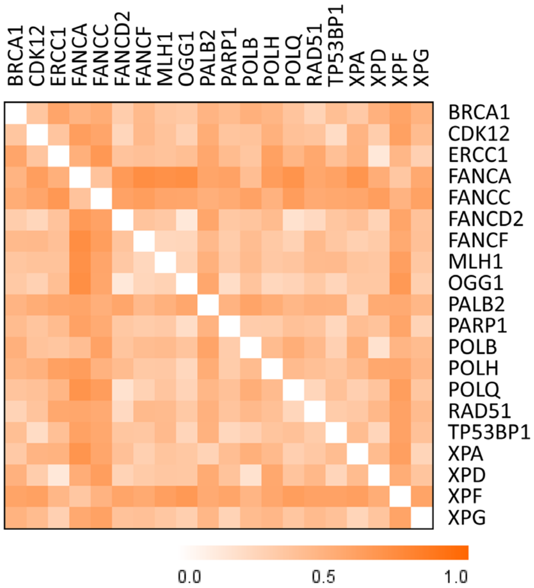

B

	MLH1	OGG1	PARP1	POLB	TP53BP1	BRCA1	RAD51	PALB2	POLQ	POLH	FANCA	FANCC	FANCD2	FANCF	XPA	XPD	XPF	XPG	ERCC1	CDK12
MLH1	1,00	0,15	0,03	-0,14	-0,16	-0,02	-0,01	-0,07	0,12	0,09	0,20	0,07	0,10	0,26	0,03	0,00	0,05	-0,10	-0,03	-0,01
OGG1	0,15	1,00	**0,41**	0,17	0,45	0,12	0,34	0,31	**0,56**	**0,41**	**0,54**	**0,52**	**0,75**	0,33	0,18	0,29	0,29	0,30	0,18	0,36
PARP1	0,03	**0,41**	1,00	0,19	0,39	0,13	0,22	**0,39**	0,23	0,31	0,28	0,28	0,16	0,19	0,02	0,12	0,16	0,21	0,01	0,05
POLB	-0,14	0,17	0,19	1,00	0,30	-0,14	0,04	0,26	-0,01	0,05	-0,11	0,31	0,11	0,10	-0,10	**0,54**	0,17	0,07	0,19	0,06
TP53BP1	-0,16	**0,45**	**0,39**	0,30	1,00	**0,43**	0,22	0,37	0,16	0,13	0,20	0,23	0,26	0,16	-0,02	**0,40**	0,22	0,08	0,14	**0,46**
BRCA1	-0,02	0,12	0,13	-0,14	0,43	1,00	-0,07	-0,04	0,13	-0,16	0,12	-0,09	-0,01	0,25	0,03	-0,03	-0,14	-0,17	-0,17	**0,41**
RAD51	-0,01	0,34	0,22	0,04	0,22	-0,07	1,00	**0,61**	**0,45**	0,22	0,23	**0,39**	**0,60**	0,02	0,31	0,02	**0,66**	0,07	-0,11	0,19
PALB2	-0,07	0,31	**0,39**	0,26	0,37	-0,04	**0,61**	1,00	0,32	0,26	0,07	0,19	0,35	0,03	-0,04	0,16	**0,75**	0,25	0,01	0,20
POLQ	0,12	**0,56**	0,23	-0,01	0,16	0,13	**0,45**	0,32	1,00	0,22	**0,43**	0,17	**0,72**	0,31	0,23	0,23	0,31	0,33	0,14	0,19
POLH	0,09	**0,41**	0,31	0,05	0,13	-0,16	0,22	0,26	0,22	1,00	0,29	0,13	0,20	0,12	0,00	-0,21	0,23	0,04	-0,24	-0,15
FANCA	0,20	**0,54**	0,28	-0,11	0,20	0,12	0,23	0,07	**0,43**	0,29	1,00	**0,45**	**0,41**	0,26	0,29	-0,10	0,03	0,00	-0,06	0,16
FANCC	0,07	**0,52**	0,28	0,31	0,23	-0,09	**0,39**	0,19	0,17	0,13	**0,45**	1,00	0,34	**0,48**	**0,45**	0,30	0,28	0,14	0,28	0,03
FANCD2	0,10	**0,75**	0,16	0,11	0,26	-0,01	**0,60**	0,35	**0,72**	0,20	**0,41**	0,34	1,00	0,08	0,18	0,29	**0,41**	0,17	0,15	**0,49**
FANCF	0,26	0,33	0,19	0,10	0,16	0,25	0,02	0,03	0,31	0,12	0,26	**0,48**	0,08	1,00	0,23	0,29	0,01	0,06	0,25	-0,04
XPA	0,03	0,18	0,02	-0,10	-0,02	0,03	0,31	-0,04	0,23	0,00	0,29	**0,45**	0,18	0,23	1,00	-0,01	0,25	0,24	-0,09	-0,08
XPD	0,00	0,29	0,12	**0,54**	0,40	-0,03	0,02	0,16	0,23	-0,21	-0,10	0,30	0,29	0,29	-0,01	1,00	0,12	0,30	**0,71**	0,27
XPF	0,05	0,29	0,16	0,17	0,22	-0,14	**0,66**	**0,75**	0,31	0,23	0,03	0,28	**0,41**	0,01	0,25	0,12	1,00	0,09	-0,07	0,15
XPG	-0,10	0,30	0,21	0,07	0,08	-0,17	0,07	0,25	0,33	0,04	0,00	0,14	0,17	0,06	0,24	0,30	0,09	1,00	**0,53**	-0,02
ERCC1	-0,03	0,18	0,01	0,19	0,14	-0,17	-0,11	0,01	0,14	-0,24	-0,06	0,28	0,15	0,25	-0,09	**0,71**	-0,07	**0,53**	1,00	0,18
CDK12	-0,01	0,36	0,05	0,06	0,46	**0,41**	0,19	0,20	0,19	-0,15	0,16	0,03	**0,49**	-0,04	-0,08	0,27	0,15	-0,02	0,18	1,00

Supplementary Figure 2: (**A**): heatmap of the correlation between single gene expression in the all the PDXs investigated. (**B**): correlations among the expression of the genes studied in all the ovarian PDXs.

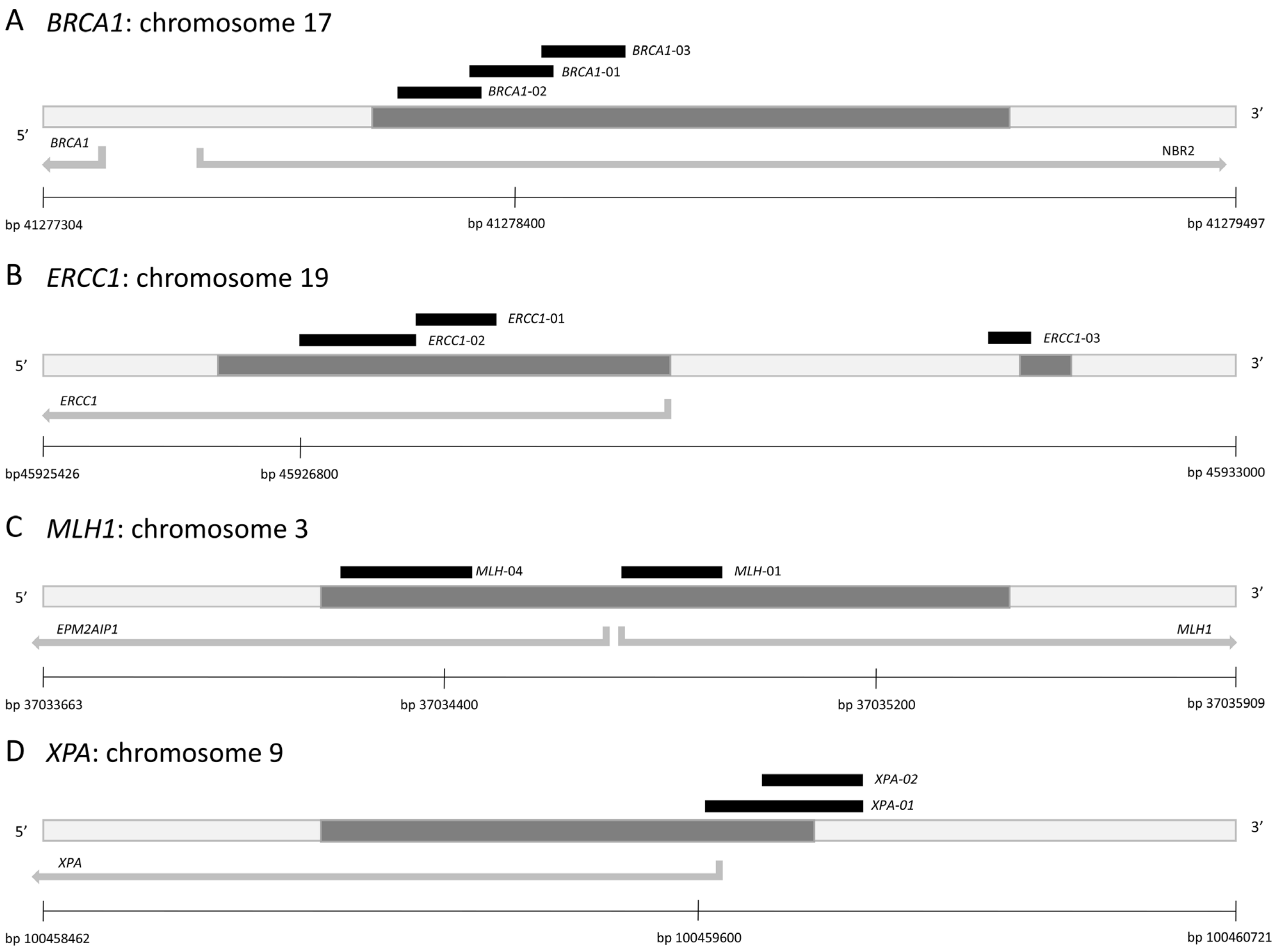

Supplementary Figure 3: Chromosomal location of the PyromarkCpG Assays (Qiagen) in the core promoter region and transcriptional regulatory sites of *BRCA1*, *ERCC1*, *MLH1* and *XPA* genes. Genomic coordinates are shown, along with the PyromarkCpG Assays (black bars) and promoter region (dark grey area). (**A**) BRCA1-01, -02 and -03 assays cover an area in BRCA1 promoter which includes 11 CpG sites. (**B**) 15 CpG sites were evaluated in ERCC1 promoter region by ERCC1-01, -02 and -03 assays. (**C**) For MLH1 two different assays were used (MLH1-01 and -04) which cover 11 CpG sites. (**D**) XPA methylation status was evaluated using XPA-01 and -02 assays in part overlapped, covering 6 CpG sites.

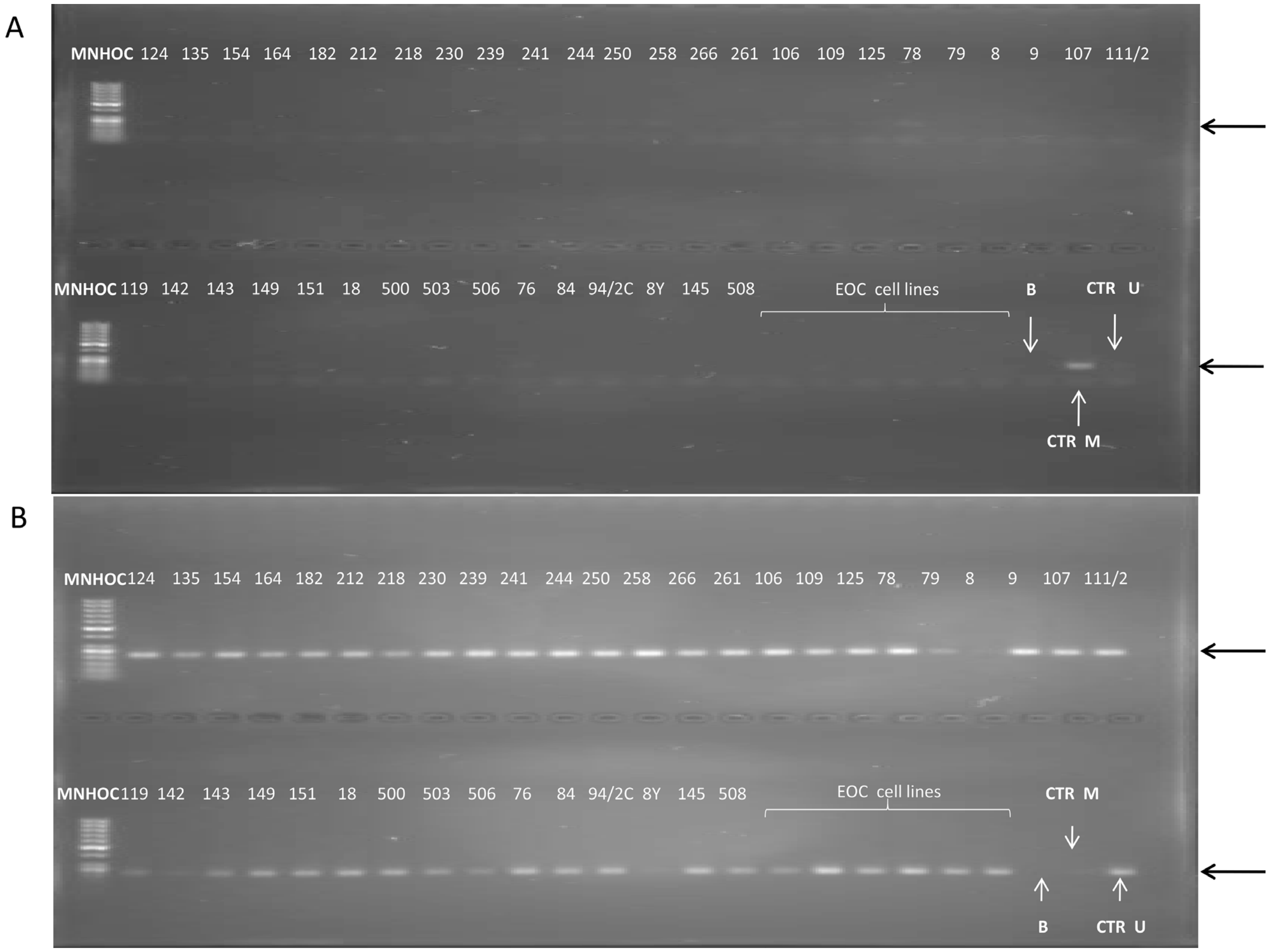

Supplementary Figure 4: Methylation status of XPG. Analysis of methylation status of *XPG* in 39 ovarian PDXs (MNHOC) and in 5 epithelial ovarian cancer cell lines by PCR with specific primers designed for methylated sequences (panel **A**) and unmethylated (panel **B**). EpiTect PCR Control DNA set (Qiagen) was used as PCR internal control (CTR M: methylated DNA and CTR U: unmethylated DNA); B: blank.

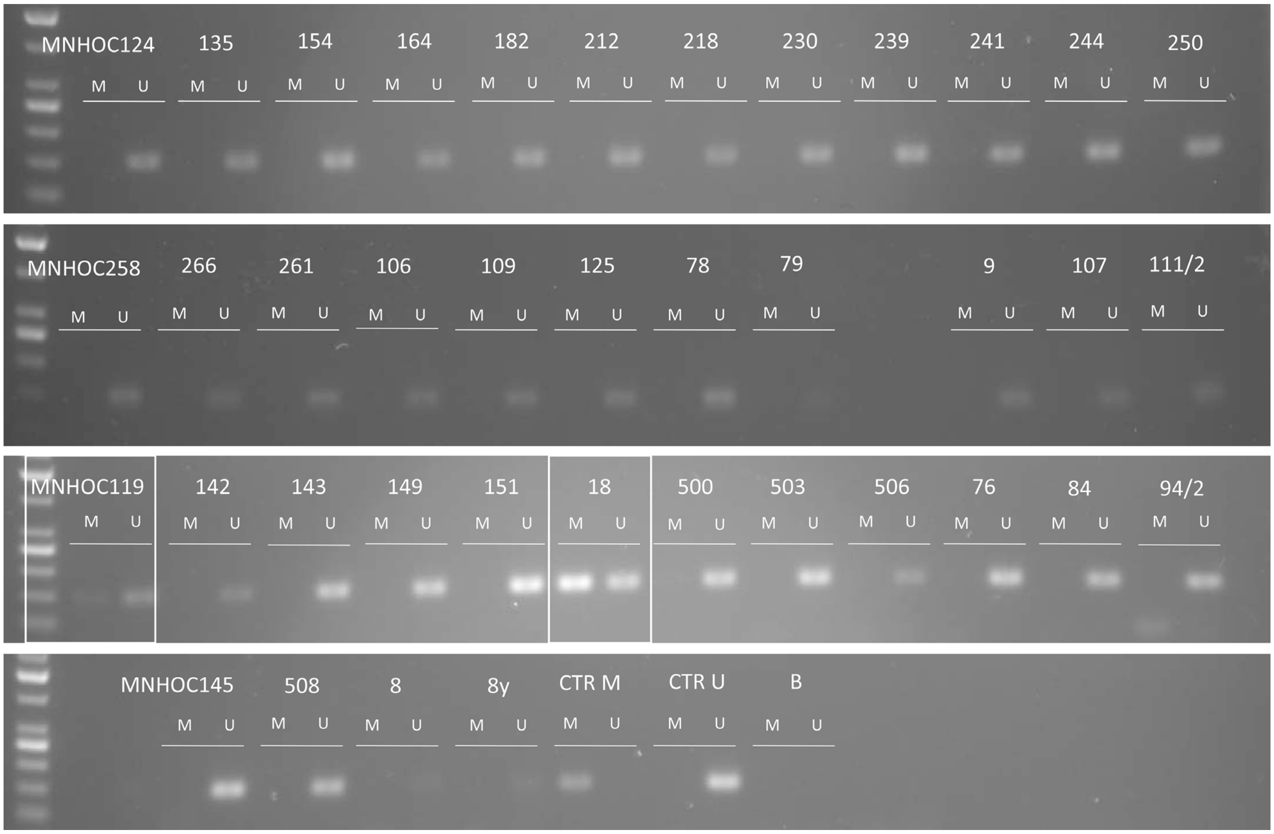

Supplementary Figure 5: Methylation pattern of FANCF. Analysis of methylation status of FANCF in 39 ovarian PDXs by PCR. Visible bands are 153bp PCR products obtained with primers specific for methylated (M) or unmethylated (U) sequences. EpiTect PCR Control DNA set (Qiagen) was used as PCR internal control (CTR M: methylated DNA and CTR U: unmethylated DNA); B: blank.
